# Perspective From the 5th International Pemphigus and Pemphigoid Foundation Scientific Conference

**DOI:** 10.3389/fmed.2018.00306

**Published:** 2018-11-08

**Authors:** Jinmin Lee, Victoria P. Werth, Russell P. Hall, Rüdiger Eming, Janet A. Fairley, David C. Fajgenbaum, Karen E. Harman, Marcel F. Jonkman, Neil J. Korman, Ralf J. Ludwig, Dedee F. Murrell, Philippe Musette, Haley B. Naik, Christian D. Sadik, Jun Yamagami, Marc L. Yale, Aimee S. Payne

**Affiliations:** ^1^Department of Dermatology, University of Pennsylvania, Philadelphia, PA, United States; ^2^Corporal Michael J. Crescenz VAMC, Philadelphia, PA, United States; ^3^Department of Dermatology, Duke University, Durham, NC, United States; ^4^Department of Dermatology and Allergology, Philipps-Universität Marburg, Marburg, Germany; ^5^Department of Dermatology, University of Iowa, Iowa City, IA, United States; ^6^Orphan Disease Center, Perelman School of Medicine, University of Pennsylvania, Philadelphia, PA, United States; ^7^Centre of Evidence Based Dermatology, University of Nottingham, Nottingham, United Kingdom; ^8^Department of Dermatology, University Medical Center Groningen, University of Groningen, Groningen, Netherlands; ^9^Department of Dermatology, University Hospitals Cleveland Medical Center, Case Western Reserve University, Cleveland, OH, United States; ^10^Department of Dermatology, Lübeck Institute of Experimental Dermatology, University of Lübeck, Lübeck, Germany; ^11^Department of Dermatology, University of New South Wales, Sydney, NSW, Australia; ^12^Department of Dermatology, Rouen University Hospital, Rouen, France; ^13^Program for Clinical Research, Department of Dermatology, University of California, San Francisco, San Francisco, CA, United States; ^14^Department of Dermatology, Allergy, and Venereology, University of Lübeck, Lübeck, Germany; ^15^Department of Dermatology, Keio University, Tokyo, Japan; ^16^International Pemphigus and Pemphigoid Foundation, Sacramento, CA, United States

**Keywords:** rituximab, Btk, FcRn, eotaxin, T-cell, fumarate, leukotriene, doxycycline

## Abstract

The 5th Scientific Conference of the International Pemphigus and Pemphigoid Foundation (IPPF), “Pemphigus and Pemphigoid: A New Era of Clinical and Translational Science” was held in Orlando, Florida, on May 15–16, 2018. Scientific sessions covered recent, ongoing, and future clinical trials in pemphigus and bullous pemphigoid, disease activity and quality of life instruments, and the IPPF Natural History Study. Furthermore, the meeting provided an opportunity to hear firsthand from patients, investigators, and industry about their experience enrolling for clinical trials.

## Introduction

Following successful meetings in Bethesda, Maryland, USA in 2001, 2005, and 2010, and Lübeck, Germany in 2017, the 5th International Pemphigus and Pemphigoid Foundation (IPPF) Scientific Conference was the first meeting to focus on clinical trials in pemphigus and pemphigoid and their methodologies. The meeting was organized by Drs. Aimee S. Payne, Victoria P. Werth, Russell P. Hall III, and IPPF Director Marc L. Yale and brought together over 175 researchers, clinicians, industry representatives, and patients representing 13 different countries to discuss the latest scientific and clinical data from technologies advancing to clinical trials in pemphigus and pemphigoid.

## What's new in pemphigus and pemphigoid clinical trials?

The first session of the meeting focused on results of ongoing and recently completed clinical trials in pemphigus, moderated by Drs. John Stanley (University of Pennsylvania, Philadelphia, PA, USA) and Ron Feldman (Emory University, Atlanta, GA, USA), as well as pemphigoid, moderated by Drs. David Woodley (University of Southern California, Los Angeles, CA, USA) and Enno Schmidt (University of Lübeck, Lübeck, Germany).

### Pemphigus

B cells are major effector cells in autoimmunity, both through autoantibody production as well as cellular tolerance mechanisms. Dr. Philippe Musette (Rouen University Hospital, Rouen, France) spoke about major advances being made in refractory pemphigus patients by using B cell depleting therapies, in particular the anti-CD20 monoclonal antibody rituximab. A recent clinical trial (NCT00784589) supports rituximab usage as a first line therapy in pemphigus patients (1). Additionally, B cell depletion in patients represents an opportunity to better understand pemphigus pathophysiology and human B cell biology. Dr. Musette reported that rituximab induces a prolonged and continuous repopulation of naive B cells with a new repertoire after the initial B cell depletion, whereas the reappearance of memory B cells is markedly delayed (2). IL-10-producing regulatory B cells are also expanded and are capable of downregulating inflammation, making them potentially important for maintenance of tolerance. This delay of B cell maturation is associated with a blockage of the auto-reactive IgM to IgG class switching process. Thus, B cell depletion induces a two-step mechanism of immunosuppression in pemphigus by eliminating the autoreactive B cells involved in the production of pathogenic IgG+ autoantibodies and by promoting the appearance of regulatory B cells that can maintain long term immune tolerance.

Inhibition of Bruton's tyrosine kinase (BTK), a protein essential for B cell development, is an appealing therapeutic strategy for pemphigus based on the dual mechanism of action to block autoantibody production as well as to quickly dampen inflammation by inhibiting B cell activation. Dr. Dedee Murrell (University of New South Wales, Sydney, Australia) reported on an open label study of the oral BTK inhibitor, PRN1008 (NCT02704429), which was previously tested in healthy volunteers (3). In the current study, safety and efficacy was demonstrated in 21 pemphigus patients with mild to moderate disease (Pemphigus Disease Area Index score of 8–39), with a disease duration between 0 and 20 years. The primary endpoint of control of disease activity after 4 weeks treatment with PRN1008 was met in 61% of patients, who were concomitantly using a daily prednisone dose between 0 and 30 mg. PRN1008 will shortly commence a large, global, placebo-controlled, randomized phase 3 study in patients with pemphigus.

Inhibition of the neonatal Fc receptor (FcRn) can promote protection from autoimmunity by reducing serum antibody levels and blocking pro-inflammatory immune pathways. Recently, a humanized IgG4 monoclonal antibody was developed that specifically inhibits FcRn function (SYNT001) (4). This antibody has been evaluated in a phase 1b open label safety, tolerability, and activity study to treat subjects with pemphigus (NCT03075904). Dr. Russell Hall (Duke University, Durham, NC, USA) presented results of the phase 1b study showing that infusion of SYNT001 in human subjects resulted in a rapid lowering of circulating levels of IgG and was safe and well-tolerated. Subjects were found to have a mean total IgG reduction of 56% by Day 30, and 5 of 7 subjects showed reduction in disease activity by day 42 (5). Further studies are ongoing to evaluate the efficacy and safety of FcRn blockade in the treatment of pemphigus.

### Pemphigoid

Dr. Karen Harman (University of Nottingham, Nottingham, UK) explained the characteristics of pragmatic and non-inferiority trials, illustrated by the BLISTER (bullous pemphigoid steroids and tetracyclines) study (ISRCTN13704604). A pragmatic trial is designed to reflect everyday clinical practice and represents the opposite end of the spectrum to an explanatory trial, performed under ideal conditions. The degree of pragmatism is measured over 9 domains using the PRECIS-2 tool. A non-inferiority trial is used to assess whether a test treatment is not clinically worse than standard care (control) by more than an acceptable predetermined margin (the non-inferiority margin) and comes from the concept of an acceptable alternative that may not be quite as effective standard care but may have other advantages such as cost, convenience or side-effects. The BLISTER study concluded that doxycycline is non-inferior to oral prednisolone for short-term disease control in bullous pemphigoid and significantly safer in the long-term (6).

Since bullous pemphigoid is characterized by a predominance of eosinophils both in the skin and in the blood, it is logical to target the eosinophil by treating with an antibody directed against eotaxin, a chemokine whose levels are elevated in the skin and blood of patients with BP. Dr. Neil Korman (Case Western Reserve University, Cleveland, OH, USA) discussed the results of a pilot phase 2a study of the safety and efficacy of bertilimumab, an anti-eotaxin-1 antibody, in the treatment of patients with bullous pemphigoid (NCT02226146). The results demonstrated that bertilimumab was safe and efficacious in the treatment of bullous pemphigoid. Despite only receiving three bertilimumab infusions and low doses of prednisone that were rapidly tapered, the nine subjects in this study showed rapid and durable improvement in disease activity with an 81% reduction in disease severity, along with a significant steroid-sparing effect. These promising preliminary findings should be followed up with larger controlled trial of longer duration.

Dr. Janet Fairley (University of Iowa, Iowa City, IA, USA) reviewed the current state of therapeutic clinical trials in bullous pemphigoid and studies of the mechanisms of lesion formation that could lead to new treatments. Currently there are no approved drugs for pemphigoid, and the advanced age of these patients and their co-morbidities have made clinical trials challenging. However, recently a number of new treatment options have been identified. Potential targets include complement, eosinophil chemotaxis and activation, polymorphonuclear cells, IgE and IgE receptors, IgG turnover, and cytokines. Several of these therapeutic strategies have shown promise in pre-clinical trials and case reports or case series. Larger controlled trials may lead to better treatments for pemphigoid patients.

## Clinical trial enrollment and outcomes

The second session of the meeting discussed data using disease-specific instruments that may help to determine clinical trial enrollment and outcomes, moderated by Drs. Katerina Patsatsi (Aristotle University School of Medicine, Thessaloniki, Greece) and Michael Hertl (Philipps-Universität Marburg, Marburg, Germany).

Dr. Jun Yamagami (Keio University, Tokyo, Japan) discussed a longitudinal study to quantify disease extent throughout the course of pemphigus and pemphigoid therapy, in order to better understand how the change in PDAI (Pemphigus Disease Area Index) or BPDAI (Bullous Pemphigoid Disease Area Index) score within the first 2 weeks from the initial treatment predicts whether the patient will require additional treatment. The study found significant change in the PDAI/BPDAI scores from baseline to 2 weeks between patients who needed additional treatment and those who did not, indicating that the ratio of PDAI/BPDAI scores could be useful as an objective parameter to determine the necessity of additional treatments.

Dr. Victoria Werth (University of Pennsylvania, Philadelphia, USA) discussed assessing the “quality” of quality of life surveys in autoimmune blistering disease. There are currently a number of skin-specific and one disease-specific autoimmune blistering disease quality of life instruments. A meta-analysis of health related quality of life in pemphigus identified at least 16 quality of life studies using 8 different instruments. It is important to determine which of the main instruments used capture the impact of disease on patients for both epidemiologic and therapeutic studies. This study sought to compare the change in three quality of life measures, including the autoimmune bullous diseases quality of life (ABQOL) questionnaire, the Dermatology Life Quality Index (DLQI), and the Skindex-29 (the latter of which has three subscores for symptoms, function, and emotion), with change in disease severity (measured in pemphigus with the Pemphigus Disease Area Index (PDAI) and Autoimmune Bullous Skin Disorder Intensity Score (ABSIS), and in bullous pemphigoid with the Bullous Pemphigoid Disease Area Index (BPDAI) and ABSIS) (7). Twenty three patients with mucosal involvement and 27 without mucosal involvement were enrolled and followed prospectively for a mean of 4.6 months. Sixty eight percent had pemphigus vulgaris, 12% had pemphigus foliaceus, and 20% had bullous pemphigoid. The Skindex-29 symptoms subscore correlated best with the change in disease severity (*r* = 0.75) relative to the ABQOL and DLQI (*r* = 0.65 and 0.68). The ABQOL had the best correlation with change in mucosal disease severity relative to Skindex-29 symptoms and DLQI (*r* = 0.77 vs. 0.66 and 0.56), and the Skindex-29 symptoms relative to ABQOL and DLQI had the best correlation with change in skin disease severity (*r* = 0.81 vs. 0.0.59 and 0.75). Overall the change in BPDAI and PDAI correlated more strongly with quality of life measurements than the ABSIS. Dr. Werth noted that the patients enrolled in the study had relatively mild disease, and higher correlations with the PDAI and BPDAI may reflect the ability to capture low disease activity accurately with these instruments.

## Patient reported outcomes

The third session of the meeting focused on patient-reported outcomes, moderated by Drs. Meng Pan (Shanghai Jiao Tong University, Shanghai, China) and Animesh Sinha (University at Buffalo, Buffalo, NY, USA).

On behalf of the International Pemphigus and Pemphigoid Foundation (IPPF), Marc Yale (IPPF, Sacramento, CA, USA) summarized the Natural History Study being conducted by the IPPF. The IPPF Natural History Study is a new patient registry sponsored by the National Organization for Rare Disorders (NORD) and the US Food and Drug Administration (FDA). This online data system collects, stores, and retrieves patient data for analysis in research studies (https://pemphigus.iamrare.org/). The IPPF Natural History Study serves to provide a convenient online platform for patients or their legally authorized representative to report cases of pemphigus and pemphigoid, conduct a prospectively-planned natural history study that will result in the most comprehensive understanding of both diseases and their progression over time, and characterize and describe the pemphigus and pemphigoid population as a whole. In addition, it also serves to assist the pemphigus and pemphigoid community with the development of recommendations for standards of care, assist researchers studying the pathophysiology of pemphigus and pemphigoid, and support the design of clinical trials that explore new pemphigus and pemphigoid treatments. The IPPF Natural History Study is designed to help the medical and research community understand illness trends, treatment outcomes, disease burden, and some important demographic information about patient age and gender. With this vital information from large numbers of pemphigus and pemphigoid patients, the IPPF can better advocate for resources to improve patient support, education and outreach, as well as accelerate research.

Subsequently, Odette Miller (pemphigus patient, New Jersey, USA), Jeff Weisgerber (pemphigus patient, North Carolina, USA), Dr. Diana Chen (Genentech, South San Francisco, CA, USA), Ann Neale (Principia Biopharma, South San Francisco, CA, USA), and Dr. Donna Culton (University of North Carolina, Chapel Hill, NC, USA) led a panel discussion to allow patients who have participated in recent trials, as well as pharmaceuticals and investigators who have enrolled advanced phase clinical trials in pemphigus and pemphigoid to share their experiences.

## Collaborative networks for rare disease research

The co-founder of the Castleman Disease Collaborative Network, Dr. David Fajgenbaum (University of Pennsylvania, Philadelphia, PA, USA) gave a keynote speech on the “collaborative network approach” that has helped to spearhead to accelerate research and drug discovery for Castleman disease (8). This information is significant because Castleman disease is one of the most common causes of paraneoplastic pemphigus, a highly deadly form of pemphigus. As rare diseases share many of the same hurdles in the way of drug discovery, there are great opportunities to leverage aspects of the collaborative network approach to make progress for pemphigus and other rare diseases.

## Clinical trials of novel cellular therapies for pemphigus

The fifth session of the meeting focused on future clinical trials in pemphigus, moderated by Drs. Annette Czernik (Icahn School of Medicine at Mount Sinai, New York, NY, USA) and Donna Culton (University of North Carolina, Chapel Hill, NC, USA).

In the pathogenesis of pemphigus, loss of immune tolerance to the major autoantigen desmoglein (Dsg) 3 is the key event leading to the production of Dsg-reactive autoantibodies. Dr. Rüdiger Eming (Philipps-Universität Marburg, Marburg, Germany) described strategies to restore immune tolerance to Dsg3 in the CD4+ T cell compartment by applying an HLA-transgenic mouse model of pemphigus. The study has shown that injecting animals with a set of immunodominant HLA-DRß1^*^04:02-binding Dsg3 peptides that are linked either to cellular carriers (splenocytes) or to defined particles (nanoparticles) prevents the induction of Dsg3-specific IgG antibodies upon Dsg3 immunization. Moreover, T cell reactivity to Dsg3 *in vitro* is markedly reduced in mice that previously received immunodominant Dsg3 peptides under tolerizing conditions. These results in the preclinical model provide the rationale for the development of a future phase I/II clinical trial to restore immune tolerance to Dsg3 in pemphigus vulgaris (PV) patients.

Adapted from a groundbreaking gene-engineered chimeric antigen receptor (CAR) T cell therapy that has led to long-lasting remissions of previously refractory B cell leukemia and lymphoma, Dsg3 chimeric autoantibody receptor T cell (DSG3-CAART) therapy has been shown to induce histologic and serologic remission of experimental pemphigus without detectable off-target toxicity in preclinical mouse models (9). Dr. Aimee Payne (University of Pennsylvania, Philadelphia, PA, USA) discussed strategies to move CAART technology forward to clinical trials in both mucosal PV, caused by antibodies to Dsg3, and in mucocutaneous PV, characterized by autoantibodies to Dsg3 and the homologous protein Dsg1. Dr. Payne reported that DSG1-CAART alone, and combined DSG1- and DSG3-CAART cells showed specific cytolysis of anti-DSG B cells, and no detectable toxicity to human skin xenografts *in vivo*. A first-in-human phase 1 clinical trial of DSG3-CAART in PV is planned to evaluate its safety and therapeutic potential.

Dr. Haley Naik (University of California San Francisco, San Francisco, CA, USA) reported on an autologous polyclonal regulatory T cell therapy as a strategy for limiting autoantibody production by augmenting immune regulation and re-establishing immune tolerance. A phase 1 open-label multicenter trial of autologous polyclonal regulatory T cells in patients with active pemphigus vulgaris and pemphigus foliaceus is being conducted toward this end (NCT03239470). In addition to clinical evaluation for safety, this study also aims to assess the presence and persistence of transferred regulatory T cells in blood and skin, alterations in the tissue immunologic milieu, and disease-specific and immunologic biomarkers in blood and skin. Safety and efficacy data generated from this study will lay the foundation for future studies using antigen-specific regulatory T cell therapy.

## Emerging therapies in clinical development for pemphigoid

The final invited speaker session covered future clinical trials in pemphigoid, moderated by Drs. Peter Marinkovich Stanford University, Stanford, CA, USA) and Soo-Chan Kim (Yonsei University, Seoul, Korea).

Dr. Marcel Jonkman (University of Groningen, Groningen, Netherlands) summarized an international survey on unmet needs in pemphigoid diseases to explore and prioritize unmet needs from the perspectives of patients, clinicians, and researchers. The priority need for patients is a quicker diagnosis, for clinicians labeling of new drugs, and for researchers more head-to-head randomized controlled trials. All surveyed groups agreed on a high need for improvement of current treatment options, and future research should focus on this unmet need.

Dr. Ralf Ludwig (University of Lübeck, Lübeck, Germany) reported on an upcoming clinical trial evaluating the safety and efficacy of dimethyl fumarate (DMF) in bullous pemphigoid, which will enroll the first patients this year in France, Germany, Poland and Turkey (DPem Trial). This trial was based on findings in preclinical animal models of epidermolysis bullosa acquisita (EBA) (10), where DMF reduced clinical disease severity in mice with already clinically manifest skin lesions. In addition to DMF, preclinical EBA models (11) have defined several new therapeutic targets, i.e., SYK (12, 13) and novel compounds (14).

Dr. Christian Sadik (University of Lübeck, Lübeck, Germany) presented the topic of the C5a-LTB_4_ axis in bullous pemphigoid diseases (15). The eicosanoid leukotriene B_4_ (LTB_4_) and the complement factor C5, the precursor of the anaphylatoxin C5a, are both abundant in lesional skin of bullous pemphigoid patients, but their significance for the pathogenesis of bullous pemphigoid or other pemphigoid diseases is still largely elusive. In his talk, Dr. Christian Sadik demonstrated preclinical results from mouse models of pemphigoid diseases pointing at a critical role of LTB_4_ in the recruitment of granulocytes to the dermal-epidermal junction (16). He discussed evidence that LTB_4_ may closely interact with C5a in the regulation of skin inflammation and that hence, inhibiting these two factors individually or in parallel may be effective in the treatment of pemphigoid diseases.

## Conclusions

The next decade offers exciting promise for an increasing number of clinical trials in pemphigus and pemphigoid as new preclinical programs advance to clinic and existing clinical stage companies apply their technologies to the treatment of pemphigus and pemphigoid. The patients, physicians, and researchers in the pemphigus and pemphigoid community remain committed to advocating for treatment options that improve the health and quality of life for pemphigus and pemphigoid patients.

## Author contributions

JL wrote the first draft of the manuscript; VW, RH, RE, JF, DF, KH, MJ, NK, RL, DM, PM, HN, CS, JY, MY, and AP wrote sections of the manuscript. All authors contributed to manuscript revision, read and approved the submitted version.

### Conflict of interest statement

The following authors have received consulting fees, equity, patent licenses, and/or grant funding from the interests listed below. RE, Topas Therapeutics. JF, argenx, Immune Pharmaceuticals. DF, Janssen Pharmaceuticals. RH, Stiefel, Syntimmune, Eli Lilly, Principia Bio, Incyte, Immune Pharmaceuticals, Hoffmann-La Roche, Pella Pharmaceuticals, Dermecular. MJ, Akari Therapeutics, Celgene, Chemocentrix, Roche/Genentech. NK, Immune Pharmaceuticals, Syntimmune, Principia Bio, Genentech. RL, Topadur, Biotest, Miltenyi Biotec, Biogen, UCB, TxCell, Incyte, Novartis, argenx, Lilly, Immungenetics. DM, Principia Bio, GSK, Novartis, Lilly, DebRA, Immunepharma, Roche. AP, Cabaletta Bio, Sanofi, Novartis, Syntimmune. CS, Akari Therapeutics. VW, Syntimmune, Novartis, Lilly, Immune Pharmaceuticals, Pharmacia, Roche/Genentech, Janssen. The remaining authors declare that the research was conducted in the absence of any commercial or financial relationships that could be construed as a potential conflict of interest.
